# Tackling imbalanced data in cybersecurity with transfer learning: a case with ROP payload detection

**DOI:** 10.1186/s42400-022-00135-8

**Published:** 2023-01-05

**Authors:** Haizhou Wang, Anoop Singhal, Peng Liu

**Affiliations:** 1 College of Information Sciences and Technology, The Pennsylvania State University, State College, USA; 2 The National Institute of Standards and Technology, Gaithersburg, USA

**Keywords:** Domain adaptation, Return-oriented programming, Imbalanced dataset

## Abstract

In recent years, deep learning gained proliferating popularity in the cybersecurity application domain, since when being compared to traditional machine learning methods, it usually involves less human efforts, produces better results, and provides better generalizability. However, the imbalanced data issue is very common in cybersecurity, which can substantially deteriorate the performance of the deep learning models. This paper introduces a transfer learning based method to tackle the imbalanced data issue in cybersecurity using return-oriented programming payload detection as a case study. We achieved 0.0290 average false positive rate, 0.9705 average F1 score and 0.9521 average detection rate on 3 different target domain programs using 2 different source domain programs, with 0 benign training data sample in the target domain. The performance improvement compared to the baseline is a trade-off between false positive rate and detection rate. Using our approach, the total number of false positives is reduced by 23.16%, and as a trade-off, the number of detected malicious samples decreases by 0.68%.

## Introduction

Deep learning has become popular in the fields of cybersecurity in recent years (Choi 2020; Berman et al. 2019), because it performs at least as good as the traditional methods do when enough high-quality data are available, and is more general and cost-effective. However, one of the challenges when applying deep learning to the cybersecurity application domain is the imbalanced data issue, which can deteriorate the performance of the deep learning models. Imbalanced data situations are quite common in cybersecurity. For example, in network intrusion detection, the amount of benign traffic is orders of magnitudes greater than malicious ones. Another example is in tackling insider threats, where the amount of normal behavior data is orders of magnitudes greater than malicious behavior. In this paper, we present a transfer learning based method to tackle the imbalanced data issue in cybersecurity using Return-Oriented Programming (ROP) payload detection as a case study. ROP is an exploit technique that can be used to perform code reuse attacks (CRA) through the Internet, which are still prominent exploit techniques today used to defeat Data Execution Prevention (DEP), especially on the legacy platforms without Address Space Layout Randomization (ASLR), because ROP payloads contain no code but only addresses. Even if ASLR is deployed, ROP attacks could still be fairly effective (Seibert et al. 2014). ROP is therefore well-studied and many methods and tools are proposed to detect ROP attacks.

In recent years, deep learning based ROP detection methods start to emerge, because they could mitigate several limitations of traditional methods. The advantages of using deep learning to detect ROP attacks include: (1) deep learning models can run independently with minimal overhead on the protected programs; (2) less human heuristics are involved to extract features or patterns; (3) a well trained deep learning model can achieve comparable detection rate (DR) and false positive rate (FPR). To the best of our knowledge, no traditional method has all the advantages mentioned above. For example, defending methods implemented at compiler (Onarlioglu et al. 2010) will change the program significantly and may cause runtime overhead; control flow integrity (CFI) based methods (Payer et al. 2015; Mashtizadeh et al. 2015) require carefully crafted fine-grained control flow graphs, which could be very costly; heuristic based methods (Chen et al. 2009; Cheng et al. 2014) may suffer from low detection rate.

Despite the potential in ROP detection, deep learning also suffers from the imbalanced data issue just as in other cybersecurity subfields. An ROP payload detection task is essentially a binary classification task: whether a piece of data is ROP payload. We observe that sometimes data with one of the two labels could be hard to prepare (Zhang et al. 2019; Li et al. 2020), and the process of generating the data with such a label becomes the bottleneck of the data preparation. Consequently, the resulting dataset could become imbalanced (or insufficient otherwise), which can substantially affect the performance of the deep learning model.

This paper introduces a model-independent transfer learning based solution to mitigate the imbalanced data issue caused by the scenario described above, using deep learning based ROP detection as a case study. The base model in this paper is DeepReturn (Li et al. 2020), which is a Convolutional Neural Network (CNN) based ROP detection model. In the case study, we have following assumptions: (1) One deep learning model is trained to protect exactly one program against ROP; (2) Enough high-quality data for at least one program is available; (3) Extremely imbalanced data for another program is available.

In our case study, we achieved 0.0290 average FPR, 0.9705 average F1 score, and 0.9521 average detection rate on 3 different target domain programs using 2 different source domain programs, with 0 benign training sample in the target domain. Compared to the baseline, the number of false positives is reduced by 23.16%, and as a trade-off, the number of detected malicious samples is reduced by 0.68%. In addition, we showed that our method is model-independent, which means that our model can be adopted regardless what kind of neural network architecture is being used. Our contributions can be summarized as follow:

- Propose a new domain adaptation based method to train a cyber-attack detection model using an extremely imbalanced dataset.
- Discuss the performance trade-offs of the proposed approach.
- Present the insights about how transfer learning helps to achieve the improved results.

Essentially, we show that our proposed method can greatly improve the practicality of a model for ROP detection.

## Background

### Return-oriented programming

**Fig. 1:**
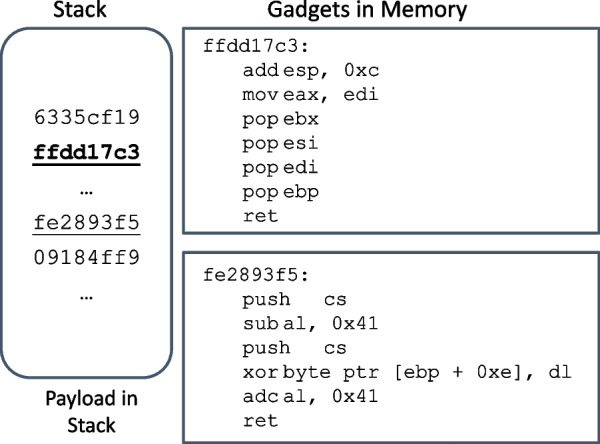
Workflow of ROP attacks

Return oriented programming (ROP) (Shacham 2007) and its variants (Snow et al. 2013; Bletsch et al. 2011; Checkoway et al. 2010) are still popular exploit methods today, which provide attackers turing-complete functionalities without inject any code. The attackers use the instruction sequences that end with an ret instruction to construct the code for their malicious purposes, which are called gadgets. In a typical ROP attack, by overwriting the return address of the executing function and loading all the addresses of gadgets needed onto the stack, the attacker will be able to execute a sequence of gadgets, which is called gadget-chain. Figure 1 shows a synthetic example of the ROP exploit process on X86 instruction set architecture (ISA). In this example, the payload arrives at the host and is loaded into a buffer on the stack. This malicious payload segment contains two addresses, 0xffdd17c3 and 0xfe2893f5, which are addresses in the code segment (i.e. .text segment) of the beginning of gadgets. If the address 0xffdd17c3 overwrites the original return address in the stack frame, it will cause the whole gadget-chain to be executed and the program will be exploited by the attacker. Since there are abundant instruction sequences (and thus gadgets) available in the memory when the program is loaded in modern operating systems, virtually ROP is turing-complete programming technique.

In practice, one important aspect of an ROP payload is the layout of the gadgets’ addresses. In the simplest scenario, the attacker needs to ensure the value stored in the %esp register is the address of the beginning of the next gadget when ret is executed. For example, pop instructions are important for the gadget layout. In X86, if the distance of the addresses of two adjacent gadgets in the memory is 4 bytes, then a pop instruction is needed to fix the offset. Since it is not common to have many pop instructions in a roll, the addresses of adjacent gadgets are usually not far away in the payload.

### Traditional ROP detection methods

The majority of the traditional ROP detection methods can be categorized into 2 kinds: heuristic-based, and CFI-based. Heuristic-based methods use heuristics and hard coded rules to find ROP gadgets. DROP (Chen et al. 2009) checks the frequency of executed return or jump instructions. kBouncer (Pappas et al. 2013) and ROPecker (Cheng et al. 2014) check indirect branches, and issue an alarm if certain abnormal patterns are found. As mentioned in “Introduction” section, these heuristics could be bypassed if the attackers know them, which results in lower detection rate.

CFI-based methods (Abadi et al. 2005; Wang and Jiang 2010; Davi et al. 2012; Zhang and Sekar 2013; Bletsch et al. 2011) use CFI to assist ROP detection. There are two disadvantages when using CFI: difficulties in building accurate fine-grained control flow graph (CFG) and causing high overhead on the program. On one hand, it is shown that building complete and accurate fine-grained CFG is very challenging (Burow et al. 2017), and in fact many works shows that attackers can circumvent CFIs using imperfect CFGs (Davi et al. 2012, 2014; Nicholas et al. 2015; Carlini and Wagner 2014). On the other hand, CFI may introduce significant overhead to the program (Mashtizadeh et al. 2015; Payer et al. 2015), which is not acceptable for performance critical services.

There are other methods that are neither heuristic-based nor CFI-based. Tanaka and Goto (2014) introduced n-ROPDetector, which checks whether a set of function addresses are presented in the payload. Since the method focuses on the payload, attackers can insert obfuscation to avoid being detected. Polychronakis and Keromytis (2011) proposed an ROP detection method based on speculative code execution, which will issue the alarm if four identified gadgets are executed. However, this could cause a high FPR, since normal instruction sequences can contain more than four gadgets, as shown by Stancill et al. (2013). There are also statistical-learning-based methods (Elsabagh et al. 2017; Pfaff et al. 2015), which usually cannot handle large datasets and need handcrafted features.

### Deep learning based ROP detection methods

Deep learning is widely used to solve many security problems, such as log anomaly detection, memory forensics, etc., where data are either widely available or easy to prepare. However, ROP attack detection, or more generally, CRA detection are relatively less popular, and one potential reason may be the difficulty when preparing the training data. From existing works, it is observed that preparing the data is the most challenging part for applying deep learning to detect ROP attacks. Li et al. (2020), Zhang et al. (2019), Chen et al. (2018). For example, Chen et al. (2018) proposed a unique data representation for traces acquired from Intel PT, which is a 2-dimensional grid data structure that can be used to training neural networks; Zhang et al. (2019) proposed a specialized fine-grained CFG and a unique way to generate malicious data.

Deep learning based ROP detection methods are showing promising results and have two major advantages: (1) usually minimal or no overhead and (2) less human efforts needed to identify heuristics and patterns. Deep learning based ROP detection methods usually have minimal overhead because deep learning models can be deployed separately. Besides, since the deep learning model can capture and extract features automatically, as long as proper representation is provided, no human effort is needed to find specific patterns. However, deep learning’s challenge is also obvious: the availability and the preparation of high quality datasets.

### Transfer learning in cybersecurity

In transfer learning, by convention, the domain where knowledge is transferred from is called **source domain**, and where knowledge is transferred to is called **target domain**. According to the survey by Pan (2009), two major categories of transfer learning are inductive learning and transductive learning. Inductive learning focuses on task knowledge transfer, whereas transductive learning focuses on data domain (representation) transfer. Notice that inductive learning assumes labels in the target domain, whereas transductive learning assumes no label in the target domain. In this paper, we focus on data domain transfer and data representation.

Recently, there are transfer learning applications in intrusion detection (Sameera 2020; Gangopadhyay et al. 2019; Singla et al. 2020), vulnerability detection (Nguyen et al. 2019; Liu et al. 2020, and IoT attack detection (Vu et al. 2020). However, none of the existing works focus using transfer learning and domain adaptation to tackle the imbalanced data issue.

### Domain adaptation

Domain adaptation is a subfield of transfer learning, which is used to solve transductive transfer learning problems. One common strategy to do domain adaptation is constructing common representation (i.e. with the same underlying distribution) for source and target domain data. This can be achieved by using a very popular metric called Mean Maximum Discrepancy (MMD) proposed by Gretton et al. (2012), which can be used to determine if sets of samples are from the same distribution. In other words, a small MMD indicates the samples are from the same distribution. Many researchers Rozantsev and Salzmann (2018), Tzeng et al. (2014), Long et al. (2015) found that a neural network can be trained using MMD as a part of the loss function to learn representation from data of both domains, so that the representation learned follows the same distribution.

## Motivation and problem statement

### Base model and data preparation process

**Fig. 2:**
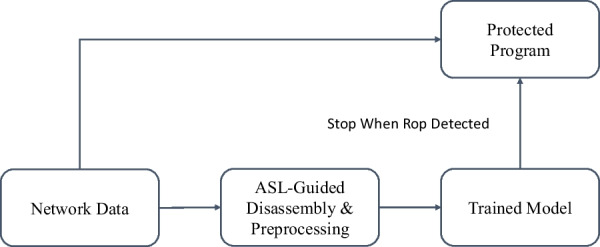
The flow diagram of DeepReturn in production

To elaborate our motivation, we first briefly explain the workflow of the base model, DeepReturn (Li et al. 2020), and the data preparation process. DeepReturn has a CNN model designed to detect ROP attacks from the network for a single program. The overall workflow of the model in production is shown in Fig. 2 To launch an ROP attack, the payloads arrive through the network, and are then sent to the victim programs. An important fact is that a malicious payload always contains addresses of gadgets in executable segments (i.e .text) of the target program, and by chaining up the gadgets found in the address space of the loaded program, the gadget-chains can be formed and executed. Other than the malicious payloads, regular data arrives through the network in a similar way, and it may or may not contain addresses of executable codes. If it does (which is a “lucky” accident), then one can also chain up a “gadget-like” instruction sequence that may or may not be executable. Therefore, the first step is trying to extract instruction sequences from the incoming network data.

If an instruction sequence can be extracted, a neural network is used to determine whether it is an actual gadget-chain (malicious) or just a “gadget-like” instruction sequence (benign), and then to determine whether the input data arrived is an ROP payload or just a piece of regular data. Therefore, the training data for the neural network are instruction sequences, where the malicious data are the gadget-chains and the benign data are the ”gadget-like” instruction sequences.

**Fig. 3:**
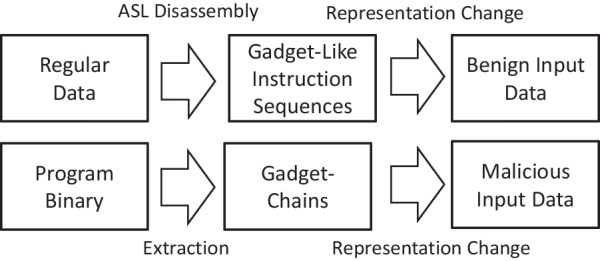
The flow diagram for the data preparation process in DeepReturn

The flow diagram of the data preparation process is shown in Fig. 3. Address Space Layout (ASL) guided disassembly is the process of chaining up the instruction sequences, which will be explained in detail in “ASL-guided disassembly” section. As shown in Fig. 3, malicious data is prepared by extracting the gadget-chains directly from the binary, and the benign data is generated by chaining up the instruction sequences using ASL guided disassembly. During the benign data generation, a piece of regular input data may or may not contain addresses of executable codes, and therefore, not all input regular data can be used to form ”gadget-like” instruction sequences. The input data which contains addresses of executable code so that it can form ”gadget-like” instruction sequences is very rare, causing the cost of generating benign data samples extremely expensive. In contrast, malicious data does not have to be generated through an actual payload. Instead, malicious samples can be easily generated by using gadget-chain generating tools, such as ROPGadget Salwan (2015).

### Issue of imbalanced data

As shown in “Base model and data preparation process” section, it is quick and cheap to generate malicious data samples; however, it is very expensive to generate benign data samples. For example, in DeepReturn, it takes 7 h to generate benign data on a cluster node with 96 CPUs for web server programs and FTP server programs. In other words, whenever the model needs to be trained or retrained, a cluster node is needed and kept running for 7 h before the training phase. In large-scale scenarios, the deep learning based method becomes less practical, because there are many programs that can suffer from ROP attacks so that many models need to be trained. Besides, it is widely agreed that programs should be kept updated for security patches, so the number of training sessions will further increase. In commercial worlds, we observe that this can be viewed as too costly to be practical.

We believe the imbalanced data is a real-world issue that may cause many deep learning based solutions to be impractical. The essence of the imbalanced data issue is the trade-off between cost and security. For example, the model maintainer can choose to train the model with the imbalanced dataset, which can cause the model to be biased. Table 1 illustrates the performance deterioration in Deep Return when 100 times more positive samples are presented in the training dataset.

**Table 1:** Effect of imbalanced dataset in DeepReturn

	1:1 Balanced	1:100 Imbalanced
False positive rate	0.399%	8.806%
F1-score	0.997	0.949

The scenario described is essentially a trade-off between cost and security: choosing imbalanced data will leave the system to be inadequately protected, whereas choosing to use balanced data can increase the cost significantly.

Therefore, mitigating the imbalanced data issue can avoid such difficult cost vs. security trade-offs. In case of the DeepReturn, if the time to generate benign data is not 7 h on a cluster node but 1 h on a personal computer, the approach will become much more practical and scalable. We observe that in this case, not all data is hard to generate, so we want to propose a method that can fully leverage the data only with the labels that are easy to generate, and requires a minimal number of hard-to-generate data.

### Model independence

Although in the base model, DeepReturn, the authors used CNN as the model backbone, we want to make our method to be model-independent. In ROP payload detection, the data samples are usually sequence data (e.g. instruction sequence, opcode sequence, etc.), so that analysts may use CNN, Recurrent Neural Networks (RNN) and their varient as the model backbone. In this paper, in addition to the base CNN model, we have also tested our method to work with RNN and hierarchical RNN proposed in DeepVSA (Guo et al. 2019).

### Problem statement

In order to address the scalability issue of the deep learning based approaches and make them more practical in the real world, we aim to solve the following problem:

Many programs may suffer from ROP attacks if a vulnerability can be used to overwrite the return address of a function to an arbitrary value. Deep learning shows its potential to detect ROP attacks effectively, but deep learning based methods often suffer from the imbalanced data issue when used to detect ROP attacks. To mitigate the effect of the imbalanced data, transfer learning may be leveraged to improve the performance of a deep learning model. The problem is whether transfer learning could be used to make the deep learning based approaches effective, scalable, and significantly more practical in the presence of an imbalanced dataset.

## Method

### ASL-guided disassembly

**Fig. 4:**
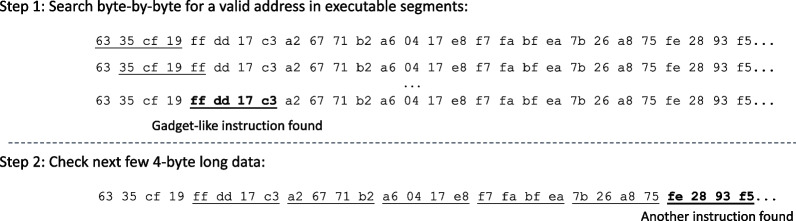
ASL-guided disassembly

This section summarizes the process of the ASL-guided disassembly to generate benign data for the training phase.

First, the reassembled network data is scanned so that the starting address of potential gadget-chains can be identified. Each byte could be the beginning of an address, and 4 consecutive bytes will be considered as an address (for x86). If an address at *n* is the start of an executable instruction sequences that end with an indirect branch, then the next 5–10 4-byte-long data (i.e. $n+4$, $n+8$...) will be evaluated to see if they are also addresses for such instruction sequences. If yes, then these instruction sequences will be chained up and let the deep learning model decide whether it is a gadget-chain.

To confirm whether an address is pointing to a valid instruction sequence, the first step is to check whether it is in the executable section (e.g. .text). Then, we will start disassembling from the address until an invalid instruction or indirect jump/call is encountered. If the disassemble is stopped because an invalid instruction is encountered, then the disassembly is stopped and this address is considered to be invalid.

Figure 4 illustrates the ASL-guided disassembly process for the payload/input shown in Fig. 1. The first step is to find a valid address that points to a potential gadget by searching through the data byte-by-byte, starting at 0x6335cf19. The first valid address is found at the byte 4, which is 0xffdd17c3. After this address is confirmed to be a gadget address, then we check if another gadget address can be found. In x86, the address takes 4 bytes, so we check the next 5 to 10 4-byte segments. Here another gadget address is found, which is 0xfe2893f5, so a data sample is identified.

To reduce the cost in this paper, for programs that would serve as source domain programs, both benign and malicious data are prepared; for programs only used as target domain programs, only malicious data and a small number of benign data samples for validation and testing are prepared.

### Base model architecture

Despite of the sequence data (i.e instruction sequences), Li et al. (2020) shows the CNN performs at least as well as the RNN does in DeepReturn, but CNN is much easier to train. In this paper, we have no motivation to change the base model, so the backbone of our model used is 1-dimensional CNN. To perform domain adaptation, there are modifications in the fully connected layers, and the details of the modification are explained in “Deep domain adaptation using mean maximum discrepancy” section.

The input data are binary instruction sequences. After the gadgets and the gadget-like instruction sequences are identified, they will be assembled back to binary. Therefore, for the neural network, the inputs are essentially byte sequences. The atomic unit is a byte, which is represented as an integer number between 0 to 255.

**Fig. 5:**
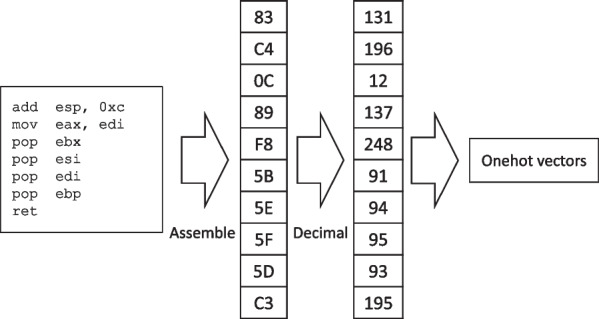
Input data representation

To eliminate the effect of the numerical relationships between each byte (i.e. 255 is larger than 0), one-hot encoding is adopted to vectorize each byte. Therefore, the final input data to be fed into the model is a sequence of one-hot vectors. Figure 5 illustrates how binary instruction sequences are processed after being identified. For example, add esp, 0xc will be assembled to 0x83 0xc4 0x0c. Then, it will be transformed to decimal numbers, which is [131, 196, 12]. Finally, the decimal numbers will be encoded to onehot.

the hidden layers follow regular CNN classifier design, so that we will not dive deep here. Overfitting issues are addressed by using dropout and early stopping. The dropout rate is 0.5, and validation data are used to stop the training early. Batch normalization is also used to stabilize the training.

**Fig. 6:**
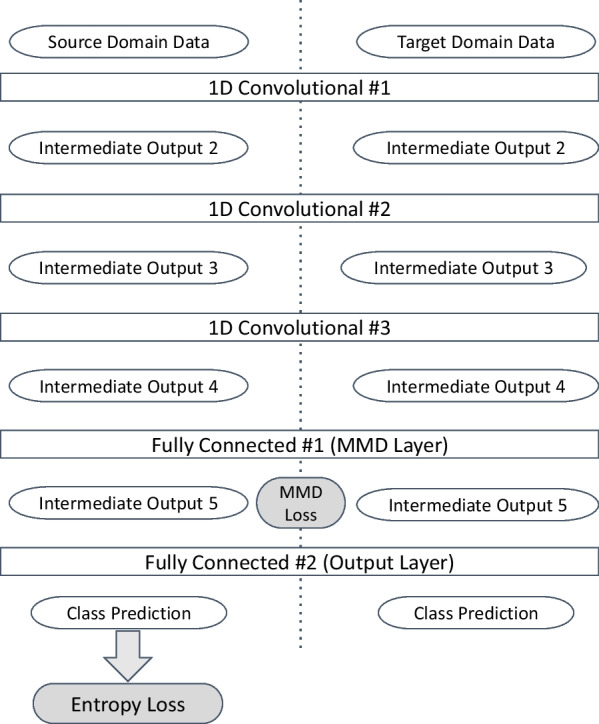
Architecture of the deep domain adaptation model

The model contains 3 convolutional layers with batch normalization, one fully-connected layer for domain adaptation, and one fully-connected layer for classification output. Figure 6 shows the details of the model.

### Deep domain adaptation using mean maximum discrepancy

Existing methods to solve the data imbalance issue have two major categories: data-based and model-based. Data-based methods sample data in dedicated ways, whereas model-based methods modify the model architectures and training processes. In our scenario, data-based methods cannot fully leverage one important advantage in our assumption mentioned in “Introduction” section: high-quality data for one program are available. Therefore, we choose a model-based method: transfer learning, to tackle the imbalanced data issue. In essence, we train a model using data from two domains (i.e. data from two different programs): source domain and target domain. The data in the source domain is balanced; whereas the data in the target domain is imbalanced. The goal is to train a model to perform well on target domain data.

One challenge for a model trained only using data from a single program to detect ROP attacks for different programs is that the gadgets available can be different, resulting the data representation to be different. Based on this observation, a subfield of transfer learning, domain adaptation fits our task very well, because domain adaptation can solve transductive learning problems where the data domains are different, but the tasks are the same.

We adopted a domain adaptation method based on MMD, which is introduced by Gretton et al. (2012). MMD can be used as a distance between two distributions, given samples retrieved from each distribution. Formally, MMD is defined in reproducing kernel Hilbert space (RKHS), denoted as *H*. Let the backbone of our neural network be $f_{\theta }(x)$, where $\theta$ are model parameters. Then, given two random variables *X* and *Y* with probability distributions *p* and *q*, respectively, the MMD is defined as:

1 $$\begin{aligned} \text {MMD} (f_{\theta }, p, q) = \Vert \mathop {\mathbb {E}}_x [f_{\theta }(X)] - \mathop {\mathbb {E}}_y [f_{\theta }(Y)] \Vert _H \end{aligned}$$

Here for $\mathop {\mathbb {E}}_x [f_{\theta }(X)]$ and $\mathop {\mathbb {E}}_y [f_{\theta }(Y)]$, we use Monte Carlo estimation, so that $\mathop {\mathbb {E}}_x [f_{\theta }(X)] = \frac{1}{m} \sum _{i=0}^{m} k(\cdot ,f_{\theta }(x_i))$. The kernel *k* used is the Gaussian kernel, which is defined as:

2 $$\begin{aligned} k(\varvec{x}, \varvec{y}) = \exp \left( -\frac{\Vert \varvec{x}-\varvec{y}\Vert ^{2}}{2 \sigma ^{2}}\right) \end{aligned}$$

We first formally define our deep domain adaptation layer, and then illustrate the whole architecture in Fig. 6. Let the source domain data be *X*, and target domain data to be *Y*, MMD then can be obtained using Equation 1, which will be one of the loss functions. In our case, random variable *X* and *Y* represent the data generated from two different programs. By minimizing the MMD, we ensure that $f_{\theta }(X)$ and $f_{\theta }(Y)$ will have similar underlying distributions, so that the classification performance could be more accurate for the target domain data. The other part of the loss function will be the regular entropy loss. To calculate the entropy loss, extra layers after the $f_{\theta }(X)$ and $f_{\theta }(Y)$ are added. Let the extra layers to be $g_{\theta '}$, then using all data samples *Z* in both domain, where $Z = {X} \cup {Y}$, the cross entropy loss can be constructed as described in “Base model architecture” section.

As shown in Fig. 6, both source and target domain data are needed during the training process. The final outputs from the source domain data and their labels will be used to construct the entropy loss; whereas the output of the MMD layer, *intermediate output 5*, will be used to calculate the MMD loss using Eq. 1. To obtain the MMD loss, the output of the MMD layer from both source domain and target domain data are needed in one training step. Note that we do not form one single loss function by summing up the cross entropy loss and MMD loss. In each training step, although the gradients of both losses with respect to the model parameters are computed in one backpropagation iteration, but the gradients are applied separately. The details are shown in “Training using no benign data in target domain” section.
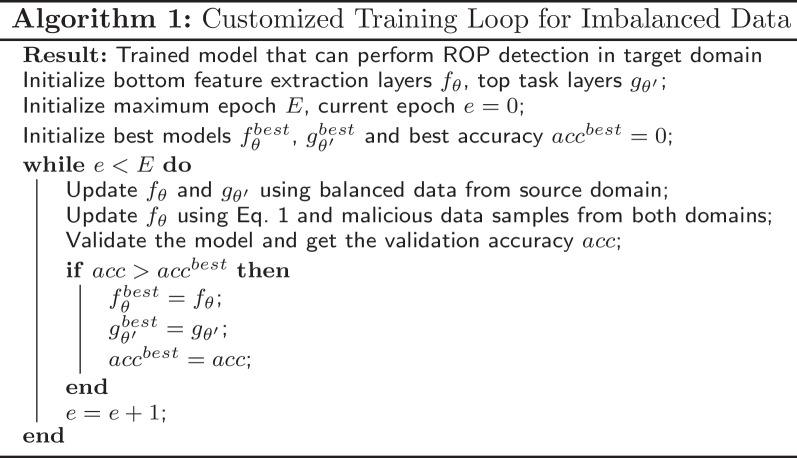


### Training using no benign data in target domain

Recall in our problem, benign data is very difficult to generate. In other words, the target domain could be extremely imbalanced, and in fact, it is preferred that **no** benign training data is needed in the target domain. However, if one only includes benign data in a dataset, the underlying distribution will be also changed. Consequently, if we still directly adopt a regular training method using MMD and entropy loss, we are inappropriately trying to create a similar distribution for two datasets with different numbers of classes. Also, different from the regular transductive learning mentioned in “Transfer learning in cybersecurity” section, the label information is known in the target domain, which should be leveraged.

Therefore, we introduce our customized training loop, which is shown in Algorithm 1. For each epoch, the entropy loss will first be calculated and minimized using the balanced data from the source domain, and then the MMD will be calculated and minimized using only malicious data in both domains. The benefits are: (1) the model will not be biased to any class for the classification task, and (2) the MMD loss will not force the intermediate outputs of the benign data to be similar to those of the malicious data.

To prevent overfitting and achieve the best test accuracy, we use early stopping. Note that the validation dataset for the early stopping purpose contains benign target domain data. Although the benign data should be avoided in the target domain, we emphasize the importance of a balanced validation dataset for an appropriate early stopping point to prevent overfitting, and will discuss the number of validation data samples required in “Evaluation” section.

In our experiments, we use Adam optimizer (Kingma and Ba 2014) with a learning rate of 0.001; the maximum epoch *E* is 25, and the batch size is 32.

## Evaluation

**Table 2:** Number of data samples used during training, validation and testing

	Train	Validation	Test
Number of M samples (source)	20,000	–	–
Number of B samples (source)	20,000	–	–
Number of M samples (target)	20,000	1750	7500
Number of B samples (target)	–	1750	1200

In our evaluations, the **baseline** is defined as the performance of a model trained using one program performing ROP detection tasks on a different program. To make the comparison fair, architectures and the training hyperparameters are as close as possible.

We first introduce the dataset. Table 2 summarizes the number of data samples used during training, validation and testing. Note that M is for malicious, and B is for benign. The maximum length of the raw instruction sequence is 128 bytes long, and gadget chains contain gadgets end with not only ret instructions but also jmp instructions.

**Table 3:** Performance of the ROP detection on target domain programs using source domain model and domain adaptation model

Source	Target	FPR		F1		DR	
		Baseline	Ours	Baseline	Ours	Baseline	Ours
proftpd	nginx	0.0217	**0.0192**	**0.9941**	0.9881	**0.9957**	0.9829
proftpd	httpd	**0.0167**	0.0183	**0.9923**	0.9843	**0.9904**	0.9754
proftpd	vsftpd	0.0392	**0.0125**	0.9484	**0.9709**	0.9142	**0.9475**
vsftpd	nginx	0.0500	**0.0475**	**0.9735**	0.9732	**0.9649**	0.9635
vsftpd	httpd	**0.0283**	0.0333	0.9508	**0.9601**	0.9151	**0.9339**
vsftpd	proftpd	0.0708	**0.0433**	**0.9733**	0.9452	**0.9713**	0.9096
Average		0.0378	**0.0290**	**0.9723**	0.9705	**0.9586**	0.9521

To generate benign data, 2 TB PDF documents image data are used as inputs for source domain programs. In the experiment in this paper, there are 20,000 benign data samples and 20,000 malicious data samples for source domain programs; there are 20,000 malicious data samples for target domain. For validation, there are 1750 benign and 1750 malicious target domain program data samples available. Then for the test, there are 1200 benign and 7500 malicious target domain program data samples. Note that for programs only used as a target domain program, only a very few number of benign data samples for validation and testing are prepared.

Accuracy is not selected as one of the performance metrics, because the test data in the target domain is extremely imbalanced. Instead, we use F1 score, false positive rate (FPR), and detection rate (DR). FPR is important because false positives are one of the most important concerns in the industry for cyber-attack detection systems.

We use 4 Internet service programs to evaluate our method. The 4 programs are proftpd 1.3.0a, vsftpd 3.03, nginx 1.4.0 and Apache httpd 2.2.18. Only proftpd 1.3.0a and vsftpd 3.03 are used as source domain programs.

In the following subsections, we will answer following research questions in the following subsections:

5.1 Can our method provide improvement, compared to directly applying source domain model to target domain data?
5.2 Can our method be adopted to other model architectures?
5.3 How is our method compared to the original model?
5.4 What is the minimum amount of validation data needed?
5.5 How is the knowledge being transferred?
5.6 What is the trade-off when comparing to traditional models?

### Can our method provide improvement across domains?

An important questions is whether our model can perform better than directly applying a model trained using one program to another program. In this evaluation, the original CNN model used in DeepReturn is adopted, and we have conducted 6 experiments using different setups, as shown in Table 3.

In Table 3, the performance metrics that have improvement with respect to the baseline are in bold. It is observed that when transfer learning is not used (i.e. baseline), the FPRs are usually higher. Among the six scenarios, there are 3 cases where the F1 score improved, 4 cases where the FPR improved, and 3 cases where the DR improved.

The best result achieved is when using proftpd as the source domain program and vsftpd as the target domain program. The improvement of the FPR is from 0.0392 to 0.0125 and the DR is from 0.9142 to 0.9475. Meanwhile, we also observe cases where the performance is not improved, such as when proftpd is the target domain program and vsftpd is the source domain program, where the DR dropped from 0.9713 to 0.9096. One observation is that whenever proftpd is used as a source domain program, the performance is already fairly good without using the domain adaptation (2 out of 3 cases). In contrast, when vsftpd is used as a source domain program, the domain adaptation seems effective and improves the performance. One potential reason for the observation is that proftpd data may include many useful features for the ROP detection.

**Table 4:** Number of true positives and false positives of domain adaptation modelk

Source	Target	TP		FP	
		Baseline	Ours	Baseline	Ours
vsftpd	nginx	**3329**	3324	60	**57**
vsftpd	httpd	3157	**3222**	**34**	40
vsftpd	proftpd	**3351**	3138	85	**52**
proftpd	nginx	**3435**	3391	26	**23**
proftpd	httpd	**3417**	3365	**20**	22
proftpd	vsftpd	3154	**3269**	47	**15**
Total		**19,843**	19,709	272	**209**

Table 4 shows the comparison between the number of detected positives (ROP attacks) and that of false positives, where the better results are in bold. According to the results, we found that the total number of false positives is reduced by more than 20%, but the total number of detected positives is only reduced by less than 0.01%. Therefore, we argue that our method can significantly improve the FPR with a small amount of trade-off on the DR.

Regarding the problem to answer, the performance of the model is largely depending on the programs in both domains. In cases where the source domain program can provide effective features for ROP detection for the target domain program, our model may be less effective; however, whenever the model trained using source domain program performs poorly on target domain program, our model performs well. In most cases, the FPRs are significantly lower.

Lastly, our domain adaptation model sometimes make things worse. To explain, it is important to remember source domain models are trained using balanced data, so from their perspectives, our method will cause a sub-effect: making the data imbalanced. Therefore, in case when two programs contain similar gadgets, this sub-effect may dominate the performance, as the source model already can do detection on the target program.

### Can our method be adopted to other model architectures?

One key feature of our method is model-independent, as introduced in “Model independence” section. To verify, we selected two different models: RNN and Hierarchical RNN proposed in DeepVSA (Guo et al. 2019). Besides, we have made the instruction sequences shorter, since RNN model usually perform poorly on long sequences.

**Table 5:** Performance on RNN and hierarchical RNN

	Source	Target	DR		FPR	
			Baseline	Ours	Baseline	Ours
RNN	proftpd	nginx	0.9800	**0.9899**	0.1292	**0.0758**
	proftpd	httpd	**0.9887**	0.9646	0.0867	**0.0717**
	proftpd	vsftpd	0.9226	**0.9675**	**0.1192**	0.1833
Hierarchical	proftpd	nginx	**0.9064**	0.7948	0.1575	**0.1033**
RNN	proftpd	httpd	**0.8623**	0.8096	0.0800	**0.0800**
	proftpd	vsftpd	**0.8446**	0.7203	0.2283	**0.0967**
Total			**0.9174**	0.8744	0.1335	**0.1018**

For each model, we have done 3 experiments in different setups. The result shows that for both models, similar to the results shown in “Can our method provide improvement across domains?” section, the FPR usually will have improvement, with little DR sacrifice. This trade-off pattern is clearly shown in the last row in Table 5, which compared the DR and FPR between baseline and proposed method in different models. The better results are in bold. In Hierarchical RNN, where the baseline is worse, this trade-off pattern is even more common. For example, although our approach achieves lower detection rate than the baseline, it reduces the FPR of vsftpd from 0.2283 to 0.0967, and reduces the FPR of nginx from 0.1575 to 0.1033.

Since we can observe similar trade-off pattern in different models, we argue that our method can be adopted to different models.

### Compared to original models trained using balanced and imbalanced data

It is also important to see the comparison to an original classification model trained using a balanced or an imbalanced dataset. Intuitively, the original model trained using balanced dataset should outperform our method, and the original model trained using imbalanced dataset should perform worse. Using FPR as the major metric, this section evaluates such scenarios in detail.

**Table 6:** False positive rate comparison with model trained using balanced data

Program	Orig. balanced	Ours	Orig. imbalanced
nginx	0.0001	0.0284	0.2201
httpd	0.0004	0.0258	0.2530
vsftpd	0.0002	0.0125	0.0857
proftpd	0.0002	0.0433	0.0904
Average	0.0003	0.0275	0.1623

The comparison is shown in Table 6. The results of models with balanced data (first column) is adopted from DeepReturn (Li et al. 2020), and since we may have multiple results for one target program (as shown in Table 3), the FPRs for our model (second column) in the tables are the average of all results for each program. In the experiments of the original model using imbalanced data, the number of negative samples to the number of positive samples is 1:100.

Compared to the original model trained using balanced data, the average FPR has increased from 0.0003 to 0.0275. The performance deterioration in FPR is very significant, because the FPR when balanced data are provided is very low. For example, the FPR in this case for proftpd reaches 0.0904.

Compared to the original model trained using imbalanced data, we can see very significant deterioration in the FPR, which is expected. Since there are 100 times more positive samples than negative ones in the training data, the trained model is biased and tends to predict positive in most of the cases. In the worst case here, the httpd, the FPR is 0.2530, so that about 1/4 of the negative test samples are misclassified.

In conclusion, the shown result follows the intuition: the original model trained using balanced dataset performs better, and the original model trained using imbalanced dataset performs worse.

### What is the minimum number of validation data needed?

As explained in “Base model and data preparation process” section, generating benign data is expensive, and should be avoided as much as possible. Therefore, one important concern is the number of validation data needed during the training phase, because some benign data samples in the target domain are needed for validation.

**Fig. 7:**
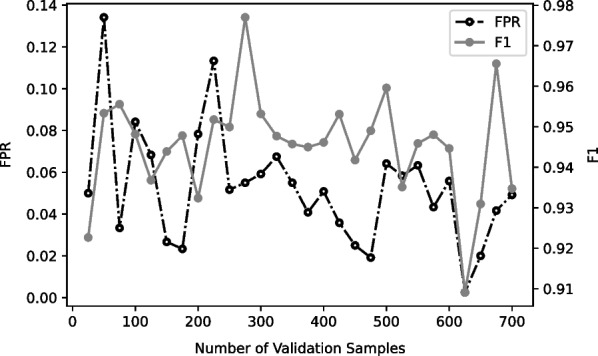
FPR & F1 versus number of validation data

Extra experiments are conducted to find an appropriate amount of validation data needed. The source domain program is proftpd and target domain program is vsfptd. The result is shown in Fig. 7. In Fig. 7, the test FPR has a decreasing trend when the number of validation data increases. However, F1 score does not has a clear trend. For example, within 100 validation data, FPR could be as high as 0.13; however, after increasing the number of validation data to over 600, the highest FPR is only about 0.03. In contrast, the F1 score does not show any trends as the validation data increases, which is still mostly between 0.92 to 0.96.

Though the FPR has a decreasing trend while the validation data size increases, the trend is far from significant and the improvement is very limited. It is important to point out that requiring few validation data does not mean no validation data needed at all. In fact, from our experiment, it is extremely important to have validation data and early stopping during the training phase. The MMD loss is very vulnerable to overfitting, and can result in very bad test performance.

### How is the knowledge being transferred?

An interesting question is whether the knowledge is actually transferred, and how the knowledge is transferred. In this section, we investigate the questions using proftpd as the source domain program and vsfptd as the target domain program.

Starting with a machine learning perspective, one important factor to consider is the MMD value. Remember MMD can be used as a distance metric for distributions, so that a small value of MMD indicates the model can learn similar representations for data from two domains. Since MMD is part of the loss function, the gradient descent algorithm can guarantee the decrease of the MMD.

Next we dig deeper into this question. We first propose two hypotheses:

**H1:** Transfer learning helps the model to capture knowledge in target domain and discard features that are not shared by two domains.
**H2:** Transfer learning will not make the model discard source domain knowledge that is useful.

To test **H1**, we first identify a sample from the target domain that is correctly classified by our model and incorrectly classified by the baseline model. Listing 1 shows the disassembly of the selected sample. By evaluating the semantics of this gadget-chain snippet, it contains many gadgets for manipulating the stack for jumping to other gadgets (e.g. sequence of pops), which could be very program-specific because of the different address space layout for different programs. Since this gadget-chain is target-domain-specific, it is not very surprising that the model completely trained using the source domain data incorrectly classifies it.
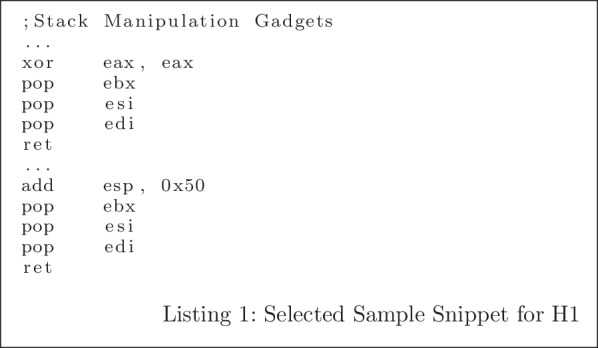


We also evaluate the uniqueness of the gadget-chain quantitatively by calculating the dissimilarity between the instruction sequences using Longest Common Subsequence (LCS) of opcodes. We first find a baseline by calculating the combination pairwise average LCS between source domain and target domain instruction sequences, which is 18.35; then we find the average LCS of the sample in Listing 1 and all other data in source domain, which is 19.42. From this result, we conclude that the selected sample shown in Listing 1 is fairly target domain specific, and we expect the extracted feature from this example using our transfer learning model and baseline model should be more different than average. The intuition is that target domain special cases should be treated specially, and our model will capture different features to make the classification correct.

The similarity between the extracted features can be measured by calculating the euclidean distances between the intermediate outputs from two models. Since the baseline model and our model are trained separately, it is not appropriate to make direct comparison between the intermediate outputs from two models. Instead, we first estimate the distance between two intermediate output spaces as baseline by averaging the combination pairwise distances of all intermediate outputs from both domains, which turns out to be 1.26. Then the average distance between the intermediate output of the selected sample and all source domain samples is calculated, which is 1.38. This result shows that compared to most of the other samples in the target domain, the intermediate output of the selected target domain sample is fairly distinct from the intermediate outputs of the source domain.

To test **H2**, we want to find two similar instruction sequences, one from each domain, and see if their intermediate outputs are similar as well. The intuition is that since the transferred model can inherit the useful features, it can extract similar features from two similar instruction sequences from different domains. Listing 2 and Listing 3 show two similar data samples (i.e. instruction sequences) from the two domains, respectively. We first show the similarity between the two gadget-chain snippets using semantic explanation. As shown in the code snippets, both gadget-chain snippets are trying to first manipulate the stack for the next gadget, and then manipulate the eax register to initialize system calls. However, since it is from different programs, we can see that the actual instructions are different, but some common gadgets can still be found.
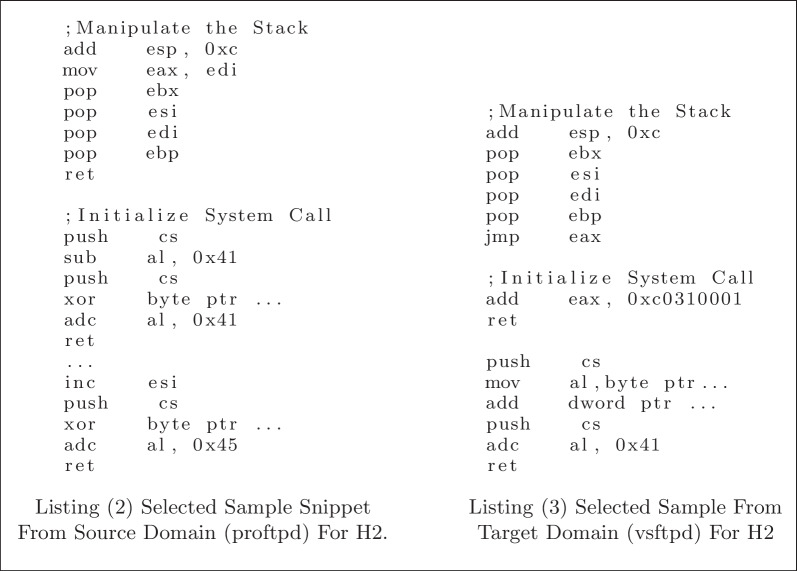


Then, we use a quantified distance measure to show the similarity. First, the baseline distance is the average euclidean distance of every possible pair of source and target domain intermediate outputs from our trained model (i.e. the combination of the set). Note that different from what has been done in H1, this time all the intermediate outputs are from our trained transfer learning model. The baseline distance is 0.0141, and the distance between the intermediate outputs of the two code snippets in Listing 2 and Listing 3 is 0.0054. The distances show that the two code snippets selected have similar intermediate outputs.

### What is the trade-off when comparing to traditional models?

As mentioned in “Traditional ROP detection methods” section, there are many existing traditional ROP detection methods. Compared to them, the deep learning based methods have two major advantages: (1) minimal or no overhead and (2) less human effort on identifying heuristics. However, the assumption for the two advantages is that the deep learning methods have comparable performances.

**Table 7:** Illustration of trade-offs between different ROP detection methods

Method	Detection performance	Overhead
DROP (Chen et al. 2009)	0% FPR	$\sim$ 500%
kBouncer (Pappas et al. 2013)	0% FPR	$\sim$ 4%
CFI (Bletsch et al. 2011)	N/A	0–5% majority $\sim$ 20% worst case
Our method	2.9% Model FPR 95.21% DR	0%

In Table 7, we have selected three traditional methods to do comparisons on different aspects, where two of them are heuristic-based, and one of them is CFI-based.

For the two heuristic-methods, DROP and kBouncer, both FPRs are 0, and the DRs are not reported. The overheads of kBouncer and DROP are about 4% and 500%, respectively. Heuristic-based methods usually have extremely low FPR, but as a trade off, their DR may not be satisfying and could have substantial overhead. Besides, heuristic-based methods may need extra human labor to craft heuristics and attributes.

CFI-based methods are considered as very accurate in detection, but the overhead cannot be avoided. In Bletsch et al. (2011), the majority of reported programs have overhead about 0–5%. Furthermore, usually the overhead varies significantly on different programs, because any compiler optimizations, obfuscations, and/or even program semantic (i.e. needs of frequent branching) will affect the overhead. As a result, the reported worst case overhead in Bletsch et al. (2011) is about 20%. Regarding the performance, although detection performance is not measured in Bletsch et al. (2011), it is mentioned in Bletsch et al. (2011) that *”the protection is only as good as the control flow graph being enforced”*, and the approach proposed in Bletsch et al. (2011) can only handle a portion of the security-relevant indirect control flow transfers. For the indirect flow transfers yet to be handled, it is stated in Bletsch et al. (2011) that they might be handled if *”the programmer or higher-level language provided more precise insight.”*

Deep learning methods usually have less overhead and require less human efforts. We use the performance of our model on target programs to compare with traditional methods. We first put a remark on the FPR, because the FPR presented is the model FPR, and the absolute majority of the inputs to the model are likely to be positive in production setup due to the ASL-Guided Disassembly procedure. Our model FPR is 2.9%, and DR is 95.21%. Regarding the overhead, since the detection system will be deployed outside the protected program, there is therefore no overhead.

## Limitation and conclusion

We identify three limitations of our approach. First, although very few, minority class data samples are still needed for validation purposes. This could make our approach impractical if the minority class samples are completely unavailable or extremely rare. The assumption of our approach is that it is very difficult, but not impossible to generate benign samples. Second, our approach requires high-quality source domain data. During the experiments, we observe that the quality of the source domain data can affect the performance substantially. Third, as illustrated in “Can our method provide improvement across domains?” section, the selection of a source domain program is important to achieve good results. However, currently we do not have a method to determine what programs are good to serve as a source domain program.

In conclusion, this paper presents a transfer learning method to mitigate the imbalanced data issue when applying deep learning in cybersecurity, using ROP payload detection as a case study. We propose a new domain adaptation based method to train a cyber-attack detection model using extremely imbalanced dataset; discuss the performance trade-offs of the proposed approach; and discuss the insights about how domain adaptation helps to achieve better results. Both strength and the limitation of our approach are discussed, and the FPR vs. DR trade-off is being identified.

## Data Availability

When certain data sharing requirements are met, the data is available upon request. Such requests should be sent to the first author of this paper.

## Abbreviations

ASL: Address space layout
ASLR: Address space layout randomization
CFG: Control flow graph
CFI: Control flow integrity
CNN: Convolutional neural network
CRA: Code reuse attack
DEP: Data execution prevention
DR: Detection rate
FPR: False positive rate
ISA: Instruction set architecture
LCS: Longest common subsequence
MMD: Mean maximum discrepancy
RKHS: Reproducing kernel Hilbert space
RNN: Recurrent neural networks
ROP: Return-oriented programming
